# Stromal interaction essential for vascular endothelial growth factor A-induced tumour growth via transforming growth factor-*β* signalling

**DOI:** 10.1038/bjc.2011.460

**Published:** 2011-11-01

**Authors:** A C Weidenaar, A ter Elst, K R Kampen, T G J Meeuwsen-de Boer, H J M de Jonge, F J G Scherpen, W F A den Dunnen, W A Kamps, E S J M de Bont

**Affiliations:** 1 Division of Pediatric Oncology/Hematology, Department of Pediatrics, Beatrix Children's Hospital, University Medical Center Groningen, University of Groningen, PO Box 30.001, Groningen 9700 RB, The Netherlands; 2 Division of Pathology, Department of Pathology and Medical Biology, University Medical Center Groningen, University of Groningen, Groningen, The Netherlands

**Keywords:** VEGFA, tumourigenesis, stroma, angiogenesis, TGF-*β*

## Abstract

**Background:**

High vascular endothelial growth factor (VEGFA) levels at the time of diagnosis confer a worse prognosis to multiple malignancies. Our aim was to investigate the role of VEGFA in promoting tumour growth through interaction with its environment.

**Methods:**

HL-60 cells were transduced with VEGFA165 or control vector using retroviral constructs. Control cells (*n*=7) or VEGFA165 cells (*n*=7) were subcutaneously injected into NOD/SCID mice. Immunohistochemistry of markers for angiogenesis (CD31) and cell proliferation (Ki67) and gene expression profiling of tumours were performed. Paracrine effects were investigated by mouse-specific cytokine arrays.

**Results:**

*In vivo* we observed a twofold increase in tumour weight when VEGFA165 was overexpressed (*P*=0.001), combined with increased angiogenesis (*P*=0.002) and enhanced tumour cell proliferation (*P*=0.001). Gene expression profiling revealed human genes involved in TGF-*β* signalling differentially expressed between both tumour groups, that is, TGFBR2 and SMAD5 were lower expressed whereas the inhibitory SMAD7 was higher expressed with VEGFA165. An increased expression of mouse-derived cytokines IFNG and interleukin 7 was found in VEGFA165 tumours, both described to induce SMAD7 expression.

**Conclusion:**

These results suggest a role for VEGFA-driven tumour growth by TGF-*β* signalling inhibition via paracrine mechanisms *in vivo*, and underscore the importance of stromal interaction in the VEGFA-induced phenotype.

The level of vascular endothelial growth factor (VEGFA) at time of diagnosis is an independent prognostic factor for treatment outcome in multiple malignancies, including (paediatric) acute myeloid leukaemia (AML) (Aguayo *et al*, 1999; de Bont *et al*, 2002). As a result, targeting VEGFA has been a subject of extensive research during the last decades.

In general, tumour growth is supported by angiogenesis, the formation of new blood vessels. An increase in microvessel density (MVD) is seen in many malignancies. In AML, enhanced bone marrow angiogenesis at diagnosis was shown, recovering to normal levels when CR was achieved (Padro *et al*, 2000; de Bont *et al*, 2001). Moreover, a correlation was found between the degree of VEGFA expression and the increase in bone marrow vascularisation at AML presentation (de Bont *et al*, 2001).

Co-expression of VEGFA and its tyrosine kinase receptors VEGFR1 and VEGFR2 has been reported previously in AML. Upon binding of its ligand, both receptors can activate several downstream pathways such as PI3-kinase and MAPK signalling, inducing cell proliferation and cell survival (Lewis *et al*, 1998; Bellamy *et al*, 1999; Grandage *et al*, 2005). Apart from autocrine effects, secreted VEGFA stimulates endothelial and stromal cells that may drive leukaemic cell proliferation via paracrine effects (Fiedler *et al*, 1997; Lee *et al*, 2007).

Although a role for VEGFA in malignant progression was implicated, the exact mechanism by how increased VEGFA levels influence tumour growth is still not completely understood. In this study a model was generated to investigate the effect of VEGFA in tumour progression. Using a human AML cell line transduced with VEGFA165 or empty vector (negative control), we were able to show that *in vitro* overexpression of VEGFA165 did not influence cell growth patterns and drug resistance, whereas *in vivo* VEGFA165 overexpression enhanced tumour outgrowth and induced the expression of host-derived cytokines. Our study suggests that VEGFA165-promoted tumour growth was mediated by a paracrine-induced inhibition of the TGF-*β* signalling pathway, and underlines the importance of targeting the tumour microenvironment.

## Materials and methods

### Cell line culture and transduction

The human AML cell line HL-60 was obtained from the ATCC (Manassas, VA, USA) in 2006. Cells have last been tested and authenticated by flow-cytometry analysis in 2009. HL-60 cells were cultured in RPMI-1640 medium supplemented with 1% penicillin/streptomycin and 10% fetal bovine serum (Hyclone, Logan, UT, USA). Retroviral supernatants were generated by cotransfection of 2 *μ*g reporter constructs pMSCV-iGFP-VEGFA165 or pMSCV-iGFP (empty vector, negative control) and 2 *μ*g packaging plasmid pCLampho into 293T cells using FuGENE HD transfection reagent (Roche, Almere, the Netherlands). HL-60 cells (5 × 10^4^) were incubated with retroviral supernatants, which were filtered through 0.45-*μ*m pore size syringe-mounted filters. Incubation was supplemented with 8 *μ*g ml^−1^ polybrene. This procedure was repeated for 2 consecutive days after which stably transduced cells were expanded. Transduction efficiency was measured by FACS analysis, which demonstrated an efficiency of 25% for cells transduced with the empty vector and 10% for cells transduced with VEGFA165. Cells were sorted on a MoFlo (DAKO Cytomation, AS, Glostrup, Denmark); after sorting the percentage of transduced cells was 90% for the empty vector (control cells) and 67% for the VEGFA165 vector (VEGFA165 cells). Cells were cultured overnight under described serum conditions before use.

### Animal study

NOD/SCID mice (kindly provided by Dr LD Shultz, The Jackson Laboratory, Bar Harbor, ME, USA) were bred and maintained at the Central Animal Facility, University of Groningen, Groningen, the Netherlands. Animals were kept under laminar flow conditions during the experiment. Our model was generated by subcutaneously injecting 10 × 10^6^ VEGFA165 cells (VEGFA165 tumour) or control cells (control tumour) into the right flank of sublethally irradiated (2 Gy) 6–8-week-old NOD/SCID mice (*n*=14). Mice were monitored for 15 days, during which tumour growth was assessed periodically. Tumour volumes were determined by external measurement according to the equation (*V*=*L* × *W*^2^) × 0.5, where *V*=volume, *L*=length and *W*=width (Tsujii *et al*, 1998). Mice were killed using cervical dislocation at day 15 and tumours were harvested. Tumours were split and either snap-frozen or fixed in formalin and embedded in paraffin for immunohistochemistry or other analyses. All procedures involving animals were performed in accordance with local ethical animal laws and policies.

### RNA extraction and real-time PCR

Total RNA from frozen tumour material was extracted with NucleoSpin RNA II kit according to the manufacturer's protocol (Macherey-Nagel, Duren, Germany). Complementary DNA was prepared at 37°C for at least 1 h in 20 *μ*l reaction mixture containing 2 *μ*g of total RNA, random hexamers (Pfizer, Capelle a/d IJssel, the Netherlands), 5 × first strand buffer, RNAsin and reverse transcriptase (Gibco BRL, Grand Island, NY, USA). Real-time PCR was performed using iQ SYBR green supermix (Bio-Rad, Hercules, CA, USA). All PCR reactions and data analysis were performed on the iCycler iQ Real-Time Detection System (Bio-Rad). Expression of genes of interest was standardised for expression of hRPL22 or m*β*-actin (arbitrary units, AU). Human-specific (h) and mouse-specific (m) primers for the reverse transcriptase PCR are listed in Supplementary Table S1. The primers were checked in NCBI blasts and did not occur in mouse genome or transcriptome. In addition, no PCR product could be detected in mouse tissue with these primers.

### ELISA and functional assay of VEGFA

Secretion of VEGFA was detected in the supernatant using commercially available ELISAs (Quantikine immunoassays, R&D Systems, Abingdon, UK) following the manufacturer's instructions. The functionality of secreted VEGFA from transduced cells was detected by adding its supernatant to endothelial cells and quantify expression of VEGFA-specific genes *EGR3*, *NUR77* and *NOR1* in endothelial cells with real-time PCR, described in detail by Liu *et al* (2003).

### Flow cytometry analysis

For BrdU incorporation, a measure of DNA-synthesis, 0.5 × 10^6^ cells were incubated for 2, 4 or 8 h with 0% or 10% fetal calf serum in presence of 1 *μ*M demecolcine (Sigma, St Louis, MO, USA) and 10 *μ*M BrdU (BD, Alphen a/d Rijn, the Netherlands), and prepared according to the BdrU-assay protocol following the manufacturer's instructions (BD); cells were neutralised for acids in 0.5 M EDTA, secondary antibody: phycoerythrin-conjugated anti-mouse (DAKO, AS, Glostrup, Denmark). Measurements with FACScalibur LSR-II (BD), data were analysed using FlowJo.

### Cellular drug resistance measurement using total cell kill assay

A total cell kill assay was performed on VEGFA165 cells and control cells using Amsacrine (0.05–2 *μ*g ml^−1^) in different concentrations in quadruplicate following former publications (Weidenaar *et al*, 2008). Optical density in the total cell kill assay is linearly related to the number of viable cells.

### Microarray analysis

RNA from 14 tumour samples was analysed using Affymetrix Human Genome U133 Plus 2.0 GeneChip (Affymetrix, Santa Clara, CA, USA). RNA quality control, cDNA labelling, microarray hybridisation, scanning, data extraction and data normalisation were performed by ServiceXS, Leiden, the Netherlands. Differentially expressed genes were identified for tumours using multivariate permutation test in Biometric Research Branch ArrayTools (BRB ArrayTools). BRB ArrayTools has been developed by the Biometric Research Branch of the US National Cancer Institute (http://linus.nci.nih.gov/BRB-ArrayTools.html). A total of 1000 permutations were completed to identify the list of probe sets. Differentially expressed probe sets were identified using a two-sample *t*-test, threshold *P*<0.001 and false discovery rate (FDR) of <0.25 was used. Gene ontology (GO) categories and KEGG pathways were determined using DAVID (Database for Annotation, Visualization and Integrated Discovery, Bioinformatics Resources 6.7, National Institute of Allergy and Infectious Diseases (NIAID), NIH, http://david.abcc.ncifcrf.gov/knowledgebase). Probe sets with a significance level of 0.001 and FDR <0.25 were used for GO analysis. Gene set expression comparisons were performed with univariate two sample *t*-test at a significance level of *P*<0.05, followed by 200 permutations. Affymetrix array results were validated using quantitative (Q)RT-PCR with human-specific primers for 11 genes of interest (Supplementary Figure S1).

### Immunohistochemical analysis for vessel density and proliferation

Frozen tumour samples were cut into 4-*μ*m sections and studied for vessel density by staining with CD31 (platelet endothelial cell adhesion molecule (PECAM)-1) as well as for proliferation by staining with Ki67. Sections were blocked for endogene peroxidase with 0.25% H_2_O_2_ and incubated with mouse-specific CD31 (BD). Subsequently, sections were incubated with secondary antibody (swine) anti-rat biotin (DAKO), amplified with (biotin) streptavidin ABComplex/HRPO (DAKO) and detected by 3-amino-9-ethylcarbazole (Sigma). After that slides were coloured with haematoxylin. Negative controls were produced using non-specific IgG as primary antibody. Sections for Ki67 were treated similarly except for the antibodies: primary monoclonal mouse anti-human Ki67 antibody (DAKO) and secondary antibody (rabbit) anti-mouse biotin (DAKO). Vessel density was assessed using light microscopy at × 50 magnification in areas of the slide containing the highest numbers of microvessels representing most intense microvasculature (hotspots). After the hotspots were identified, total number of vessels per selected image was counted at × 400 magnification. At least four hotspots were counted for each section. Proliferation was evaluated by the percentage of Ki67 positive tumour cells in four hotspot areas (selected at × 50 magnification): the selected image was divided into four areas, and the percentage of Ki67-positive tumour cells was estimated for all four areas at × 400 magnification. The mean of the four estimates was considered the count of that hotspot. Stainings were evaluated by two investigators who had no knowledge of tumour characteristics. Variability between investigators for vessel count was *ρ*=0.993 and for proliferation count was *ρ*=0.996. The mean of the two independent counts was considered to be the final measurement for each counting field and hotspot.

### Cytokine array

In VEGFA165 (*n*=4) and control (*n*=4) tumours 40 mouse-specific cytokine levels were determined by using proteome profiler mouse cytokine array panel A kit (R&D Systems) according to manufacturer's instructions (400 *μ*g protein of each sample was added). Spot densities were quantified with Scanalyze software (http://rana.lbl.gov/EisenSoftware.htm) and exported to Microsoft Excel. Spot densities were corrected for individual background to diminish interarray variances. To assess the mouse specificity of the cytokine array, protein extracts of the human HL-60 cell line and of the mouse MS5 cell line were applied to the cytokine array. As expected, a strong signal was found for the MS5 mouse cells, and no signal could be detected for the HL-60 control cells (Supplementary Figure S2).

### Statistical analysis

Wilcoxon signed-rank test was used to compare the growth of both cell lines. Tumour volumes between VEGFA165 tumours and control tumours were determined using the Student's *t*-test. Differences between tumour volumes at indicated time points were calculated using the Student's paired *t*-test. Correlation between expression of VEGFA165, tumour volume, tumour weight, percentage of cell proliferation and MVD were calculated using Spearman's correlation. Mann–Whitney *U*-test was used for comparison of vessel and proliferation counts, QRT-PCR as well as mouse-specific cytokines differentially expressed within VEGFA165 tumours and control tumours. *P*<0.05 was considered significant.

## Results

### Functional VEGFA165 overexpression in HL-60 cells

**Figure 1:**
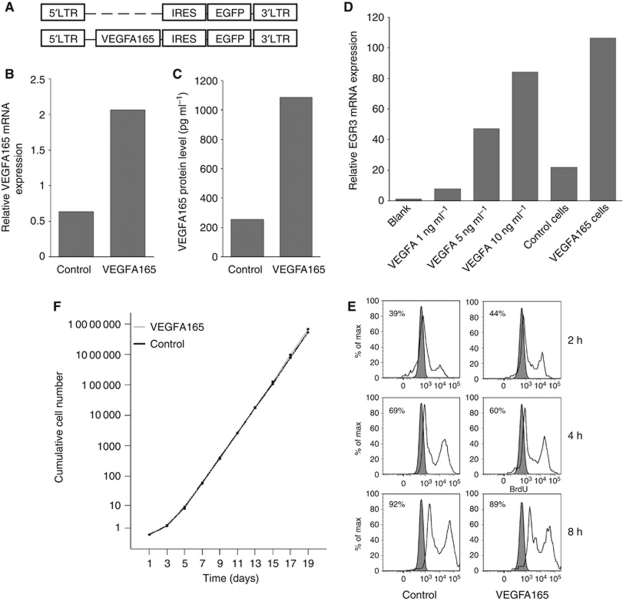
Effect of VEGFA165 overexpression *in vitro*. (**A**) Schematic representation of the retroviral constructs that were used in this study. (**B**) Relative mRNA expression of VEGFA165 in control cells compared with VEGFA165 cells. (**C**) Protein expression of VEGFA165 in control cells compared with VEGFA165 cells: control cells 254.3 pg ml^−1^, VEGFA165 cells 1085.5 pg ml^−1^. (**D**) Functionality of the produced VEGFA by transduced cells. Recombinant VEGFA induces the expression of EGR3 in ECs in a dose-dependent way (1 ng ml^−1^: 7.8 AU, 5 ng ml^−1^: 47.3 AU, 10 ng ml^−1^: 84.3 AU). Supernatant of transduced cells contained functional VEGFA, indicated by the expression of EGR3. The VEGFA165 cells showed a higher expression of EGR3 than the control cells, demonstrating that VEGFA165 cells produce more functional VEGFA (control cells: 22.0 AU; VEGFA165 cells: 106.5 AU). Similar graphs were seen when investigating the expression of NUR77 and NOR1. (**E**) BdrU incorporation of control cells and VEGFA165 cells cultured in serum-free conditions. After 2, 4 and 8 h cells were harvested and the percentage of cells that incorporated BrdU was analysed by flow cytometry. Percentage of BrdU-incorporating cells: after 2 h 39% *vs* 44% after 4 h 69% *vs* 60% after 8 h 92% *vs* 89%. Background staining in the absence of BrdU was ∼1%. Similar graphs were seen when cells were cultured with 10% fetal calf serum. (**F**) No significant difference in cell growth of VEGFA165 cells compared with control cells was seen when cells were cultured with 10% fetal calf serum (*P*=0.169, Wilcoxon signed-rank test).

We transduced HL-60 cells with VEGFA165 (retroviral vectors schematically depicted in Figure 1A). VEGFA165 mRNA expression and secreted protein levels were 3–4-fold upregulated in VEGFA165 cells compared with control cells (Figure 1B and C). To test the functionality of the produced VEGFA165, conditioned medium of VEGFA165-transduced cells or control cells was added to endothelial cells (HUVECs) and the mRNA expression of a VEGFA responsive gene *EGR3* was measured. A fivefold upregulation of EGR3 was detected in HUVEC cells after incubation with conditioned medium of VEGFA165-transduced cells compared with conditioned medium of control cells (Figure 1D). FACS analysis showed KDR expression in VEGFA165-transduced cells similar to control cells. *In vitro* cell proliferation and cell growth was similar in both cell lines (Figures 1E and F) and no difference in drug resistance was observed between VEGFA165 cells and control cells (data not shown).

### Overexpression of VEGFA165 results in increased angiogenesis and cell proliferation in a s.c. xenograft mouse model

**Figure 2:**
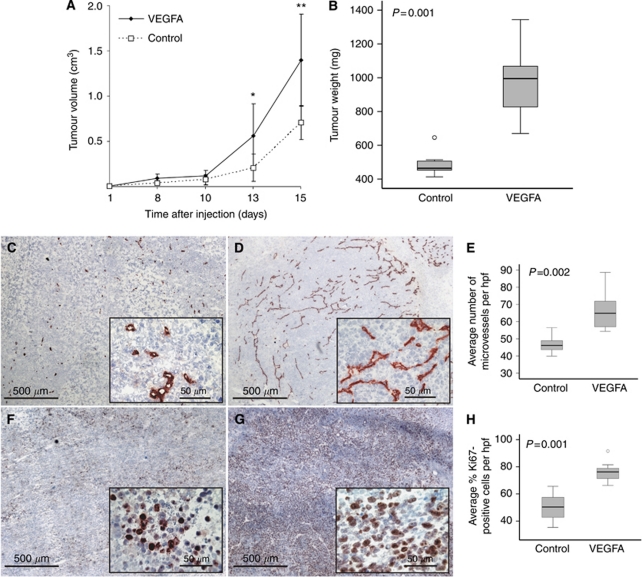
Effect of VEGFA165 overexpression *in vivo*. (**A**) Tumour volumes were assessed periodically. At day 13 and day 15 a significant difference was seen. Values expressed are the means±s.e.m. of seven xenografts per group. ^*^P<0.05, ^**^P<0.01. (**B**) Tumour weight of tumours derived from VEGFA165 cells was significantly increased compared with tumour weight of control tumours at day 15 (*P*=0.001). Median weight of VEGFA165 tumours: 995 mg (range 670–1344), control tumours: 464 mg (range 413–646). (**C**) CD31 staining of a VEGFA165 tumour and (**D**) a control tumour. (**E**) VEGFA165 tumours showed a significantly (*P*=0.002) higher MVD compared with the control tumours. Median MVD control tumours: 46.1 per field (range 39.9–56.5); median MVD VEGFA165 tumours: 64.9 vessels per field (range 54.4–88.6). (**F**) Ki67 staining of a VEGFA165 tumour and (**G**) control tumour. (**H**) A significantly (*P*=0.001) higher percentage of proliferating cells was found in the VEGFA165 tumours compared with the control tumours. Median percentage of proliferating cells control tumours: 50.3% (range 35.4–65.6); median percentage of proliferating cells VEGFA165 tumours: 76.3% (range 66.3–91.6). A representative photograph from each group is shown (original magnification × 50, with inset × 400 magnification).

To investigate the role of VEGFA165 overexpression *in vivo*, we subcutaneously inoculated VEGFA165 cells or control cells in mice. Tumour volume of VEGFA165 tumours increased more rapidly than control tumours, with a significant difference at day 13 and 15 (*P*<0.05 and *P*<0.01 respectively, Figure 2A). When tumour-bearing mice were killed at day 15 tumour weight of VEGFA165 tumours (median weight 995 mg, range 670–1344) was significantly (*P*=0.001) increased compared with the control tumours (median weight 464 mg, range 413–646) (Figure 2B). MVD in VEGFA165 tumours (median MVD 64.9 vessels per hpf, range 54.4–88.6) was significantly (*P*=0.002) enhanced compared with control tumours (median MVD 46.1 vessels per hpf, range 39.9–56.5), and a significant (*P*=0.001) increase in tumour cell proliferation fraction in VEGFA165 tumours was found (median: 76.3%, range 66.3–91.6) compared with control tumours (median: 50.3%, range 35.4–65.6) (Figure 2C–H). Interestingly, tumour weight was significantly correlated to MVD (*ρ*=0.566, *P*=0.044) and percentage of proliferating cells (*ρ*=0.722, *P*=0.004). In contrast to *in vitro* results, *in vivo* a clear phenotype could be appreciated as increased angiogenesis and tumour cell proliferation was evident.

### Distinct gene expression profiles related to VEGFA165 overexpression *in vivo*

To obtain a more detailed understanding of the phenotype within the tumour cells overexpressing VEGFA165, mRNA of VEGFA165 tumours and control tumours was isolated, amplified and hybridised to Affymetrix human U133 Plus 2.0 GeneChips. Class comparison analysis revealed 761 probe sets to be differentially expressed between the two tumour groups; 242 probe sets were higher expressed in VEGFA165 tumours whereas 519 probe sets were lower expressed in these tumours (complete list shown in Supplementary Table S2). As expected, VEGFA was significantly higher expressed in the VEGFA165 tumours compared with control tumours (*P*<1 × 10^−5^).

Subsequently, GO analysis of the upregulated probe sets in VEGFA165 tumours revealed enrichment for the process of ‘angiogenesis’ (GO:0001525, *P*=0.03) in accordance with the immunohistochemical staining for MVD. The GO term ‘cell death’ (GO: 0008219, *P*=0.02) was enriched in the downregulated probe sets of the VEGFA165 tumours, underscoring the observed increase in tumour growth and cell proliferation (complete list shown in Supplementary Table S3).

The list of up and downregulated genes in VEGFA165 tumours include known genes involved in neoplasia (e.g. *S100A8* involved in leukaemia; *S100A9* in prostate carcinoma; *BCL9*, *CCND1* and *CTNNB1*) (Hermani *et al*, 2006; Mani *et al*, 2009; Nicolas *et al*, 2011; Siapati *et al*, 2011). In addition, the cytokine receptors *IL4R* and *IL7R* were found to be higher expressed in VEGFA165 tumours implicating the possibility of paracrine effects.

Notable, genes involved in TGF-*β* signalling (*SOS1*, *SOS2*, *SMAD5*, *LTBP3*, *TGFBR2*, *IRF7* and *CREBZF*) were found to be significantly (*P*<0.01) downregulated in the VEGFA165 tumours. In addition, SMAD7, a negative modulator of TGF-*β* signalling, was found to be upregulated in VEGFA165 tumours. Three other genes involved in the TGF-*β* signalling pathway were also found to be significantly (*P*<0.05) differentially expressed (*SMAD3*, *IRF7*, *SMURF2*).

To validate these results QRT-PCR with human-specific primers was performed for 11 genes of interest (*hVEGFA*, *hCREBZF*, *hLTBP3*, *hIL4R*, *hIL7R*, *hSOS1*, *hSOS2*, *hSMAD3*, *hSMAD7*, *hSMURF2* and *hTGFBR2*). Differential expression of these 11 genes could be confirmed using QRT-PCR (Supplementary Figure S1). Therefore, we conclude that the genes involved in the TGF-*β* signalling pathway are derived from human cells within the tumours.

### Paracrine mechanisms have important roles in VEGFA165 tumours

**Figure 3:**
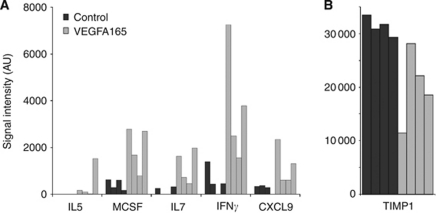
Expression of mouse-specific cytokines. Mouse-specific cytokines significantly (*P*=0.03) differentially expressed between the VEGFA165 tumours (*n*=4) and control tumours (*n*=4), showing five cytokines higher expressed (**A**) and one cytokine lower expressed in the VEGFA165 overexpressing tumours (**B**).

We hypothesised that the *in vivo* growth benefit of high VEGFA165 levels occurs via interaction with its (micro)environment. In the VEGFA165 tumours mouse-specific *VEGFR2* mRNA was twofold upregulated (*P*=0.001, Supplementary Figure S1), the main receptor for VEGFA signalling. Using mouse-specific cytokine arrays (tested for mouse specificity, Supplementary Figure S2), we assessed whether the increased tumour growth would be reflected in altered mouse-derived cytokine expression. Five mouse-specific cytokines were significantly (*P*=0.03) higher expressed in VEGFA165 tumours compared with control tumours: interleukin 5 (IL5), IL7, chemokine (C-X-C motif) ligand 9 (CXCL9, MIG), colony-stimulating factor 1 (macrophage) (MCSF) and interferon*γ* (IFNG) (Figure 3A). Tissue metallopeptidase inhibitor 1 (TIMP1) was found to be significantly (*P*=0.03) lower expressed in VEGFA165 tumours compared with control tumours (Figure 3B). Together these data show that stroma-derived (i.e. mouse-derived) cytokines are regulated in tumours overexpressing VEGFA, suggesting that VEGFA exerts its effect on tumour growth via a paracrine loop.

## Discussion

**Figure 4:**
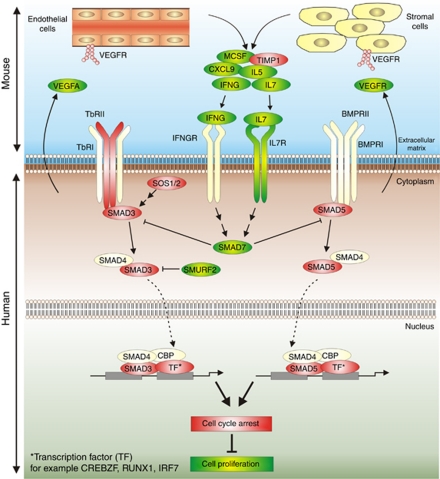
Proposed mechanism for VEGFA165 induced tumour growth. VEGFA production from the (human) tumour cells results in an increased fraction of host-derived stromal and endothelial cells. These (mouse) host-derived supportive cells produce a variety of cytokines that act on the (human) tumour cells. As a result, genes involved in the TGF-*β* signalling pathway are differentially expressed, inducing the inhibition of TGF-*β* signalling. This, in turn, results in decreased cell cycle arrest, which may explain the increased growth of VEGFA overexpression in tumour. The colours represent higher expressed (green) and lower expressed (red) genes or cytokines within the VEGFA165 tumours; stimulatory effect; indirect stimulatory effect; --∣ inhibitory effect; - - > translocation.

In this study we demonstrated that VEGFA165 overexpression significantly enhanced tumour progression *in vivo*, accompanied by a higher percentage of proliferating tumour cells and increased angiogenesis. We hypothesise that the enhanced tumour cell proliferation is accompanied by inhibition of the TGF-*β* signalling pathway in the (human) tumour cells via various stromal-derived mouse cytokines such as IL7 and IFNG (Figure 4).

High VEGFA levels predict a poor prognosis in multiple malignancies, including haematological malignancies. In our study, *in vitro* induction of high VEGFA165 levels did not alter the proliferative status of VEGFA165 cells compared with control cells despite the fact that HL-60 cells express VEGFR2 and the possibility of an autocrine loop has been described (Weidenaar *et al*, 2008). In contrast, overexpression of VEGFA165 *in vivo* resulted in an increased tumour growth and higher levels of proliferating cells.

Our data show that tumours overexpressing VEGFA165 were characterised by enhanced vessel outgrowth. An increase in MVD is seen in many malignancies, including haematological malignancies, with VEGFA as a key player (Carmeliet and Jain, 2000; Padro *et al*, 2000; de Bont *et al*, 2001). Vessels are known for their supply of oxygen and nutrients to the tumour cells, but also for excretion of various endothelial-derived growth factors of potential benefit for tumour cells (Fiedler *et al*, 1997; Dankbar *et al*, 2000). Our results show that endothelial and/or stromal cells within the tumour express higher levels of mouse-specific cytokines (MCSF, IFNG, IL5, IL7 and CXCL9) in VEGFA165 tumours compared with control tumours (Figure 4). From the literature it is known that exposure of human endothelial cells to VEGFA resulted in an increased production of cytokines, including MCSF (Fiedler *et al*, 1997; Bellamy *et al*, 1999; Dankbar *et al*, 2000). These results suggest that the effect of VEGFA165 is stromal dependent.

New is that within the human tumour cells genes involved in the TGF-*β* signalling pathway were found to be differentially expressed between VEGFA165 tumours and control tumours. The TGF-*β* signalling pathway is a negative regulator of cell cycle progression, resulting in a cell cycle arrest via transcription of TGF-*β*-responsive genes. TGFBR2, SMAD3, SMAD5, SOS1 and SOS2 were found to be lower expressed in VEGFA165 tumours compared with control tumours. In addition SMAD7 was found to be higher expressed in VEGFA165 tumours compared with control tumours. SMAD7 inhibits TGF-*β* signalling by preventing the activation of other SMADs (Kleeff *et al*, 1999). These results suggest that the block in cell cycle progression is decreased via downregulation of TGF-*β*-responsive genes, resulting in cell proliferation. This is in line with our observation that VEGFA165 tumours showed an increased fraction of proliferating cells.

As no proliferative advantage was seen for the VEGFA165 cells *in vitro* we hypothesised that the TGF-*β* signalling pathway is a result of the interaction with environment. Gene expression analysis revealed that nearly all of the genes involved in the TGF-*β* signalling pathway were not differentially expressed between the VEGFA165 HL-60 cell lines and control HL-60 cell lines. Thus, our results are in concordance with the knowledge that stromal support is essential in tumour growth *in vivo* (Ayala *et al*, 2009).

Alterations in the TGF-*β* signalling pathway are found in human malignancies, including haematological malignancies (Elliott and Blobe, 2005; Dong and Blobe, 2006). Proposed mechanisms for the reduced TGF-*β* signalling include a decrease in TGFBR2 expression or an increase in SMAD7 expression (Kim *et al*, 1999; Kleeff *et al*, 1999). In our study, tumours overexpressing VEGFA165 showed lower expression of TGFBR2 and higher expression of SMAD7, suggesting a resistance to the TGF-*β*-induced cell cycle arrest. The expression of SMAD7 can be regulated by various genes and cytokines, for example, IFNG and IL7 (Ulloa *et al*, 1999; Huang *et al*, 2002). Our data revealed that, in response to VEGFA expression by the tumour cells, these host-derived cytokines were upregulated. In addition, our microarray data showed that (human) IL7R was upregulated in the VEGFA165 tumours. Together, these data suggest that VEGFA165 induces the expression of host-derived growth factors and cytokines, which in turn induces inhibition of the TGF-*β* signalling pathway (Figure 4).

In conclusion, we show that VEGFA165 significantly enhances tumour growth and tumour angiogenesis. We demonstrated that tumour-derived VEGFA resulted in enhanced tumour cell proliferation possibly by a paracrine inhibition of TGF-*β* signalling within the tumour. These results indicate that the microenvironment is essential for VEGFA-induced tumour growth. Combining conventional therapeutic strategies with drugs targeting the tumour environment may be of benefit for tumour treatment.
