# Supplementary figures and images for: Stromal interaction essential for vascular endothelial growth factor A-induced tumour growth via transforming growth factor-β signalling
Source: Br J Cancer. 2011 Nov 1;105(12):1856–63. doi: 10.1038/bjc.2011.460 (PMC3251883; doi:10.1038/bjc.2011.460)

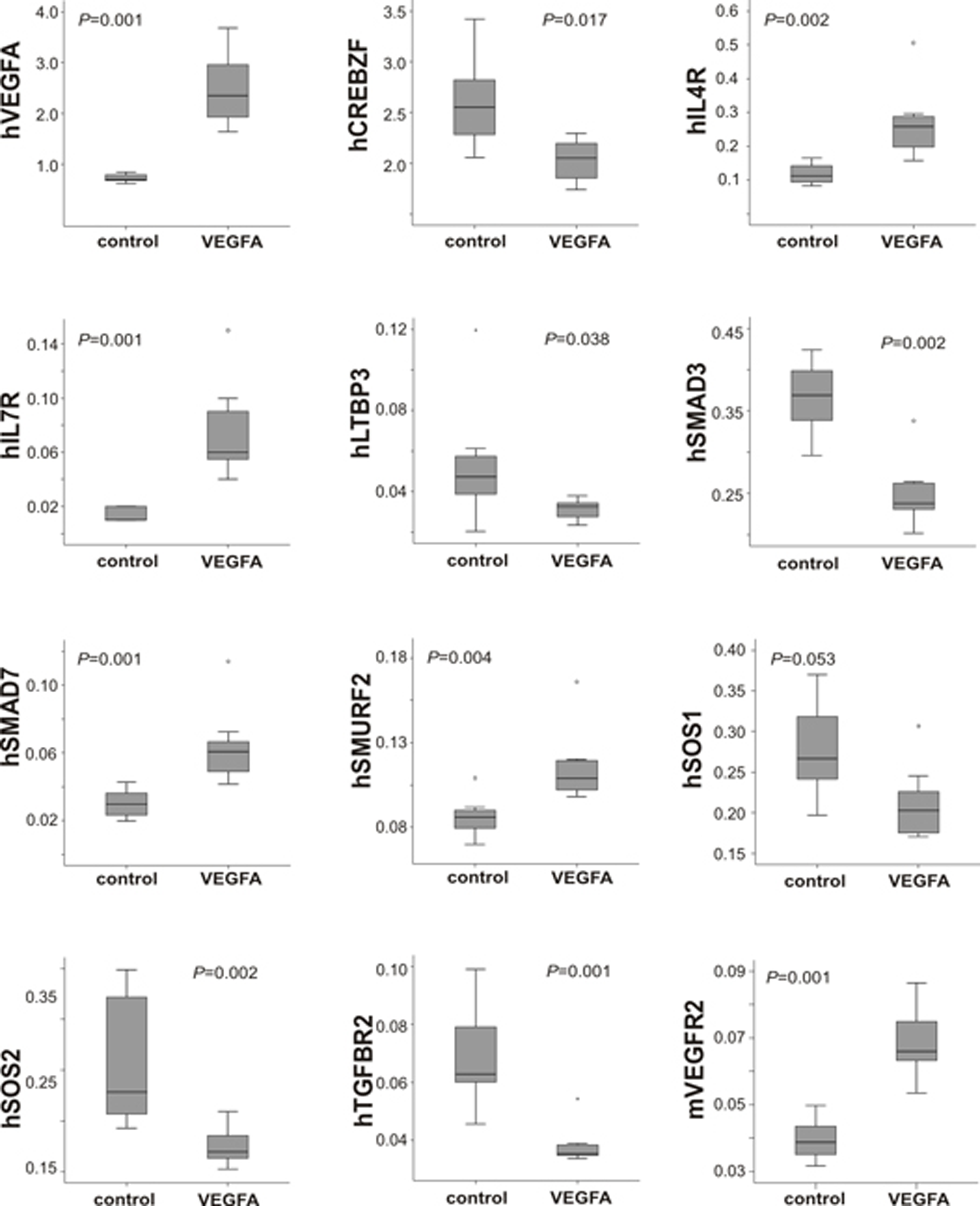

Supplement: Supplementary Figure S1 [file bjc2011460x1.tif]

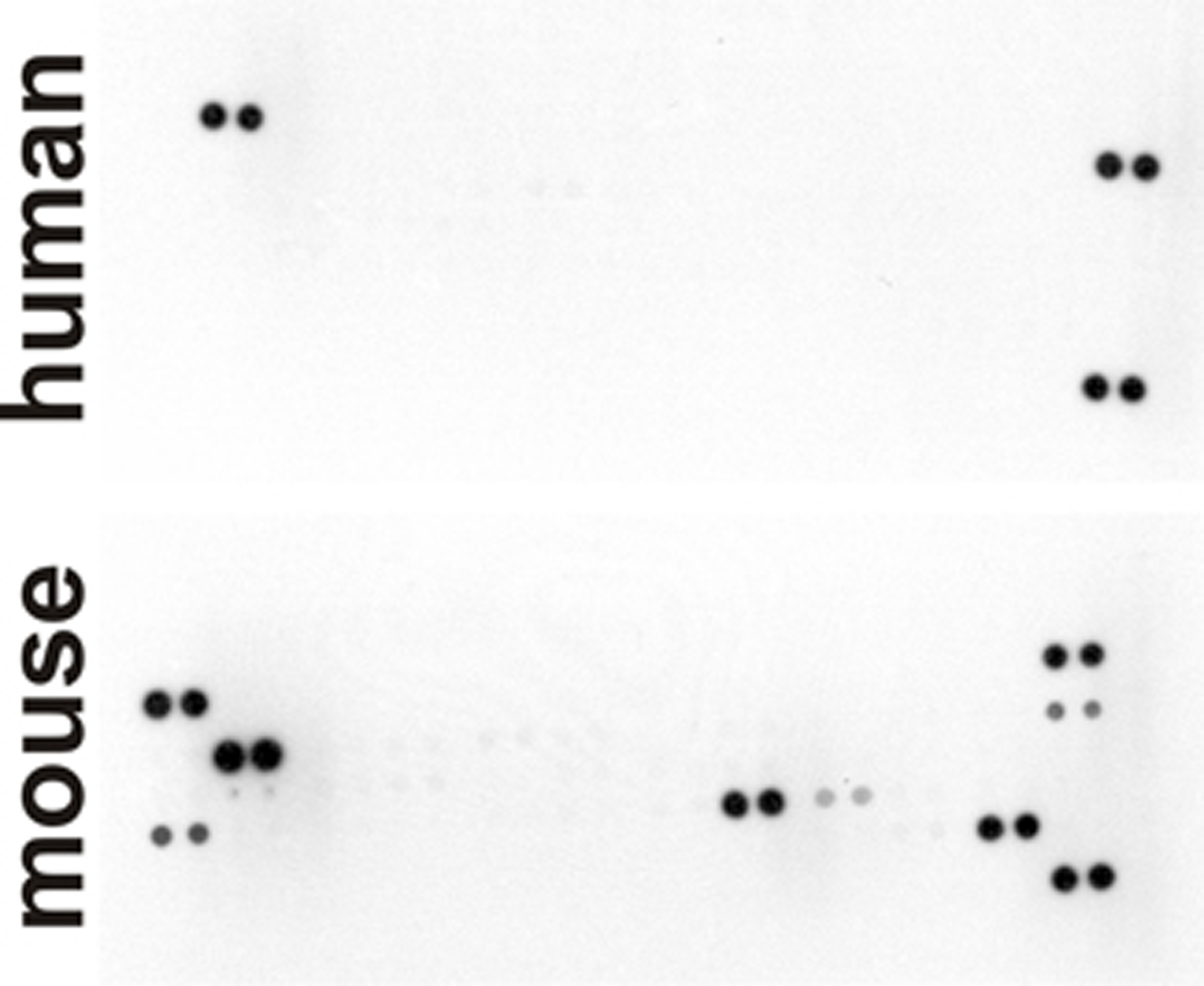

Supplement: Supplementary Figure S2 [file bjc2011460x2.tif]
