# Supplementary material for: Stromal interaction essential for vascular endothelial growth factor A-induced tumour growth via transforming growth factor-β signalling
Source: Br J Cancer. 2011 Nov 1;105(12):1856–63. doi: 10.1038/bjc.2011.460 (PMC3251883; doi:10.1038/bjc.2011.460)
Supplement: Supplementary Figure and Table Legends [file bjc2011460x6.doc]

**Supplementary data:**

**Table S1: Human-specific (h) and mouse-specific (m) primers for RT-PCR.**

**Table S2: Probes differentially expressed between the VEGFA165 tumours (n=7) and control tumours (n=7) *P=*0.05.** The list is sorted for the ratio control/VEGF: genes with a ratio <1 are higher expressed in the VEGFA165 tumours whereas a ratio >1 responds to a decreased expression in the VEGFA165 tumours.

**Table S3: Enrichment of GO terms among differentially expressed probes between the VEGFA165 tumours (n=7) and control tumours (n=7), *P=*0.05.** Genes with a significant difference of *P<*0.001 were uploaded in DAVID.

**Figure S1: Genes differentially expressed between the VEGFA165 tumours (n=7) and control tumours (n=7).** Relative mRNA expression of twelve genes of interest in control tumour cells compared with VEGFA165 tumour cells.

**Figure S2: Expression of Mouse-Specific Cytokines.** Mouse specificity was verified by adding protein extracts of mouse cell line MS5 or human cell line HL-60 to the mouse-specific cytokine array. Positive controls are shown on both blots. A strong signal was found for the MS5 mouse cells, and no signal could be detected for the HL-60 control cells.
